# An Observation of Racial and Gender Disparities in Congestive Heart Failure Admissions Using the National Inpatient Sample

**DOI:** 10.7759/cureus.10914

**Published:** 2020-10-12

**Authors:** Varun Tandon, Bryan Stringer, Chad Conner, Andre Gabriel, Byomesh Tripathi, Kathir Balakumaran, Kai Chen

**Affiliations:** 1 Cardiology, University of Arizona College of Medicine, Phoenix, USA; 2 Internal Medicine, University of Connecticut Health, Farmington, USA; 3 Cardiology, West Virginia University, Morgantown, USA; 4 Cardiology, University Hospitals, Cleveland, USA; 5 Cardiology, University of Connecticut Health, Farmington, USA

**Keywords:** cardiology, congestive heart failure, healthcare disparities

## Abstract

Background

Congestive heart failure (CHF) is a frequent cause of inpatient admissions in the United States. The purpose of this study was to analyze the racial and gender disparities that occur in CHF admissions and determine the impact of these disparities on medical expenditure.

Methods

We analyzed the National Inpatient Sample (NIS) database from 2009 to 2014 for patients with a primary discharge diagnosis of CHF, and further stratified the cohort on the basis of race and sex. Multivariate analysis was performed to identify the association between CHF and total charges along with other variables such as mortality, length of stay (LOS), and number of procedures.

Results

There were a total of 5,491,050 admissions with a primary diagnosis of CHF from 977,850 in 2009 to 901,425 in 2014. Females accounted for 49.7%. Total charges for CHF admission were highest in Asians at an average cost of $59,668. African Americans had the lowest mortality rate at 1.75%, however, they also had an average age of admission of 63.47 years, compared to Caucasian at 76.76 (p<0.05). Total charges for males were $42,920 and $36,744 for females (p <0.05). Males also had more procedures at 1.16 vs 0.98 for females (p <0.05). Elixhauser mortality score was higher in males than females at 5.95 vs 5.42 (p <0.05).

Conclusion

Healthcare disparities exist in CHF admissions in both contexts of race and gender. Further studies are required to pinpoint the source of these differences not only to address mortality but also expenditure costs.

## Introduction

Heart failure is a leading cause of hospitalizations and readmissions in the United States [1,2]. According to the Centers for Disease Control and Prevention (CDC), there were over 2 million hospitalizations for congestive heart failure (CHF) in 2016 with an estimated cost of 32.7 billion dollars [3]. This represents a significant contribution to healthcare expenditure in the United States that exceeds 3.5 trillion dollars according to the Center for Medicare and Medicaid Services (CMS) [4]. The prevalence of CHF is expected to continue to increase by 46% from 2012 to 2030 [5]. This increasing prevalence of heart failure will only add to the burden of an already expensive US healthcare system. Research has been done to investigate factors that contribute to excess cost with increased emphasis on factors such as healthcare disparities.

Healthcare disparities are defined as differences in the quality of health care that are not due to access-related factors or clinical needs, preferences, and appropriateness of interventions according to the institute of health [6]. Previous research has shown that many healthcare disparities are associated with excess cost in racial minorities, which include African American, Asian American, Pacific Islander, Native American, and Hispanic. The total estimated cost of these disparities is over 1 trillion dollars [7]. This represents a significant contribution to the overall cost of healthcare in the United States and indicates an area that can be improved significantly.

The reasons for healthcare disparities are complex. It involves patient, provider, and system-specific factors [8]. Patient-specific factors include their genetics in addition to their behaviors. For example, African American and Hispanic populations have higher rates of diabetes mellitus and hypertension, impacting their incidence of CHF, in addition to co-morbidities that impact health outcomes [9]. Provider-specific factors include unintentional biases and varying degrees of sensitivity to patient-specific cultural backgrounds. One reason for this could be explained by their own cultural background [10]. System factors include access to healthcare, insurance coverage, and infrastructure limitations [8]. Some of these factors have been addressed including an increased number of Americans with health insurance under the Affordable Healthcare Act legislation [11]. Given that healthcare disparities have contributed vastly to the cost of the healthcare system, further research needs to be done to better understand these factors and the impact they have on individual patients and the overall cost of healthcare in the United States.

Our study aimed to identify differences in inpatient mortality, total charges, and other variables among those admitted for CHF exacerbation. The rationale behind the focus on heart failure is mainly due to its high prevalence as well as the CMS focus on reducing 30-day readmission rates. We hope to highlight the financial impact of racial and sex disparities in healthcare to inspire further research and interventions to close this gap in the future.

## Materials and methods

Data source

Data was queried via the National Inpatient Sample (NIS) developed in part by the Healthcare Cost and Utilization Project (HCUP). The NIS database is one of the largest publicly available all-payer databases in the United States. The dataset per year represents approximately 35 million hospital admissions nationally.

Study population

A retrospective observational analysis was done by means of the NIS database from the year 2009 to 2014. Clinical Classification Software (CCS) code 108 was used to identify a primary diagnosis code representing CHF for the hospitalization. This code includes both populations of systolic and diastolic heart failure in order to study any patient with an active heart failure admission. Patients with CCS codes of 108 as a non-primary category code were excluded as such to remove the possibility of a non-active issue during hospitalization.

Variables

Patients diagnosed with active CHF as the principal diagnosis during hospitalization were then stratified by their race and sex. Within each race and sex, we identified the incidence, in-hospital mortality, and age. Total hospital charges, total number of procedures during hospitalization (NPR), and length of stay (LOS) were also analyzed. We additionally identified the income quartile based on the patient’s residential zip-code. Income by quartile is a scale where a score of 1 represents income from 0-25th percentile, 2 equals 26th to 50th percentiles, 3 equals 51st to 75th and 4 equals 76th to 100th percentiles. Furthermore, insurance types were analyzed in each race. In-hospital mortality scores were based on the Elixhauser Comorbidity Index, which is a dichotomous method of categorizing comorbidities of patients based on the International Classification of Disease (ICD) codes. Comorbidities were established using the Elixhauser determined comorbidities.

Statistical analysis

Statistical analyses were performed using SAS® software, version 9.4 (SAS Institute, Cary, North Carolina). Weights provided by HCUP were applied to generate national estimates. Categorical variables were analyzed using Chi-square (X2) analysis and continuous variables were tested using analysis of variance (ANOVA). For further delineation of analysis, multivariate regression was also used to determine inter-racial discrepancies and disparities. The level of statistical significance (α) was chosen as 5%.

## Results

**Figure 1:**
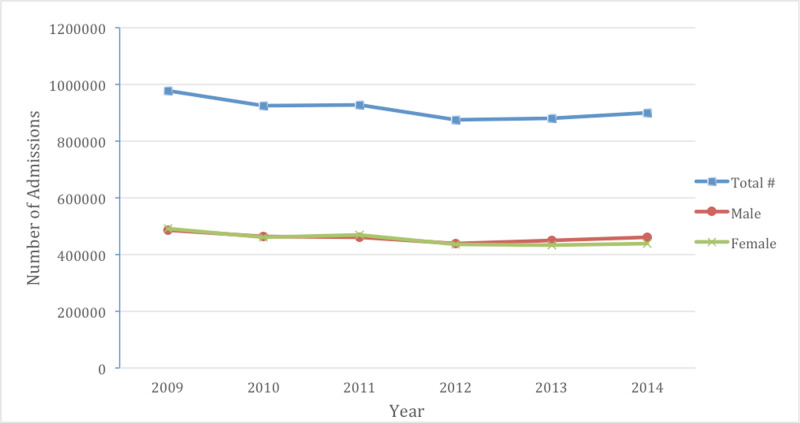
Total number of congestive heart failure (CHF) admissions by year A decrease in overall CHF admissions was noted from 2009 to 2014. This same decrease was also noted between genders with males have more admissions for CHF in 2014 than females.

**Figure 2:**
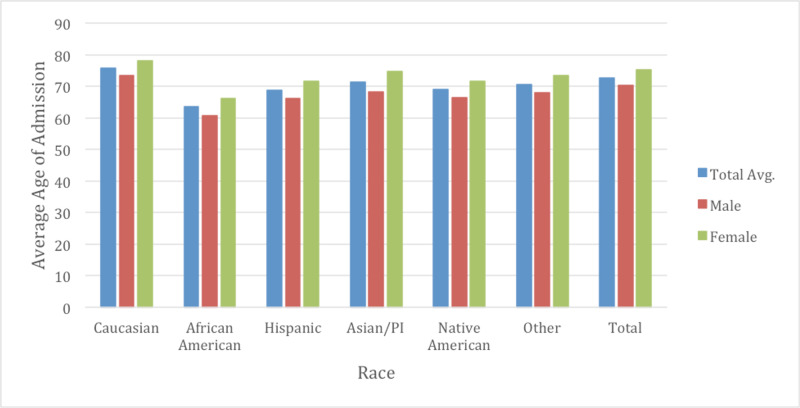
Average age of congestive heart failure (CHF) admission by race and gender The average total age of CHF admission that we noted was 72.70 years. The total average female being 75.12 and for males being 70.30 (p<0.05). On multivariate analysis, the differences in age between each race was noted to be statically significant. Additionally, for each race on gender subgroup analysis, the differences between each gender was noted to be statistically significant (p<0.05).

In this retrospective study of data, 5,491,050 patients were identified from the NIS database between 2009 and 2014 who were admitted with CHF as the primary diagnosis code. Over the course of the study, there was a slight overall decrease in CHF admissions per year from 977,850 in 2009 to 901,425 in 2014 (Figure 1). Overall, women comprised 49.7% of CHF admissions between 2009 and 2014. Caucasians constituted a majority of CHF admissions at 68.4%. This was followed by African Americans at 19.2%, Hispanics at 7.6%, Asians at 1.8%, and Native Americans at 0.6%. The average age of patients with CHF admissions was relatively stable over time: in 2009 the mean age was 72.93 years vs 72.16 years in 2014, with women being older than men across all races (Figure 2). Furthermore, the average mortality rate for CHF admissions amongst all races was 3.09%. The average LOS did not change significantly (5.23 days in 2009 vs 5.24 days in 2014). Nonetheless, the average total cost charged was noted to increase over time: from $35,572 in 2009 to $44,316 in 2014. 

Based on race, Native Americans had the highest percentage of females presenting with CHF at 50.14%. The lowest was the race deemed “Other” at 47.80% followed by Hispanics at 48.06%. The proportion of females within the Caucasians was 49.78% (p <0.05).

**Figure 3:**
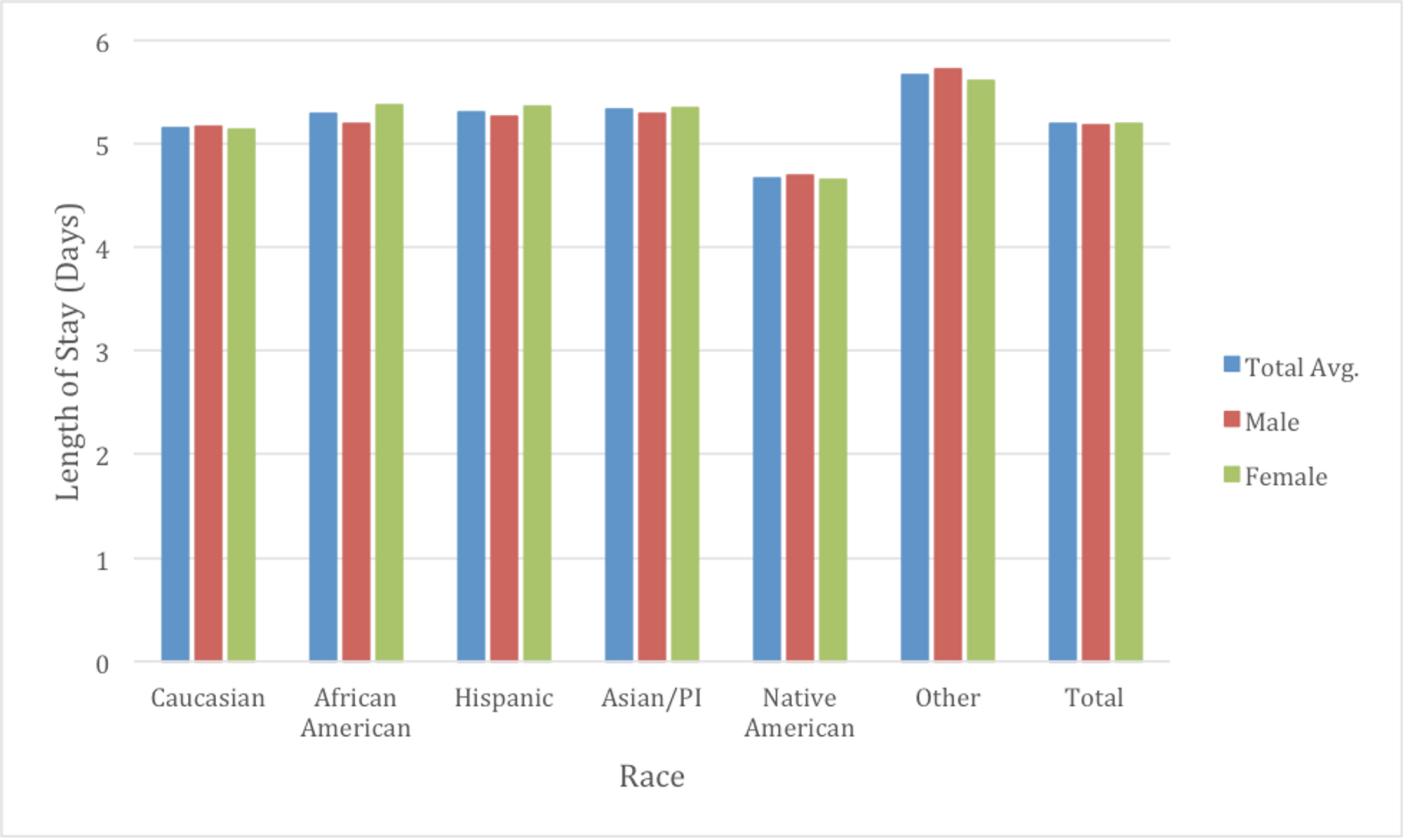
Length of stay by race and gender Overall the average length of stay we noted was 5.19 days. Males had an average of 5.18 days while females were 5.20 days (p<0.05). Native Americans were noted to have the least number of days spent in hospital for congestive heart failure (CHF) admissions. The differences between average length of stay between races was noted to be statistically significant (p<0.05). Additionally, on gender subgroup analysis, the differences between each gender was noted to be statistically significant (p<0.05) only for African Americans and Hispanics.

**Figure 4:**
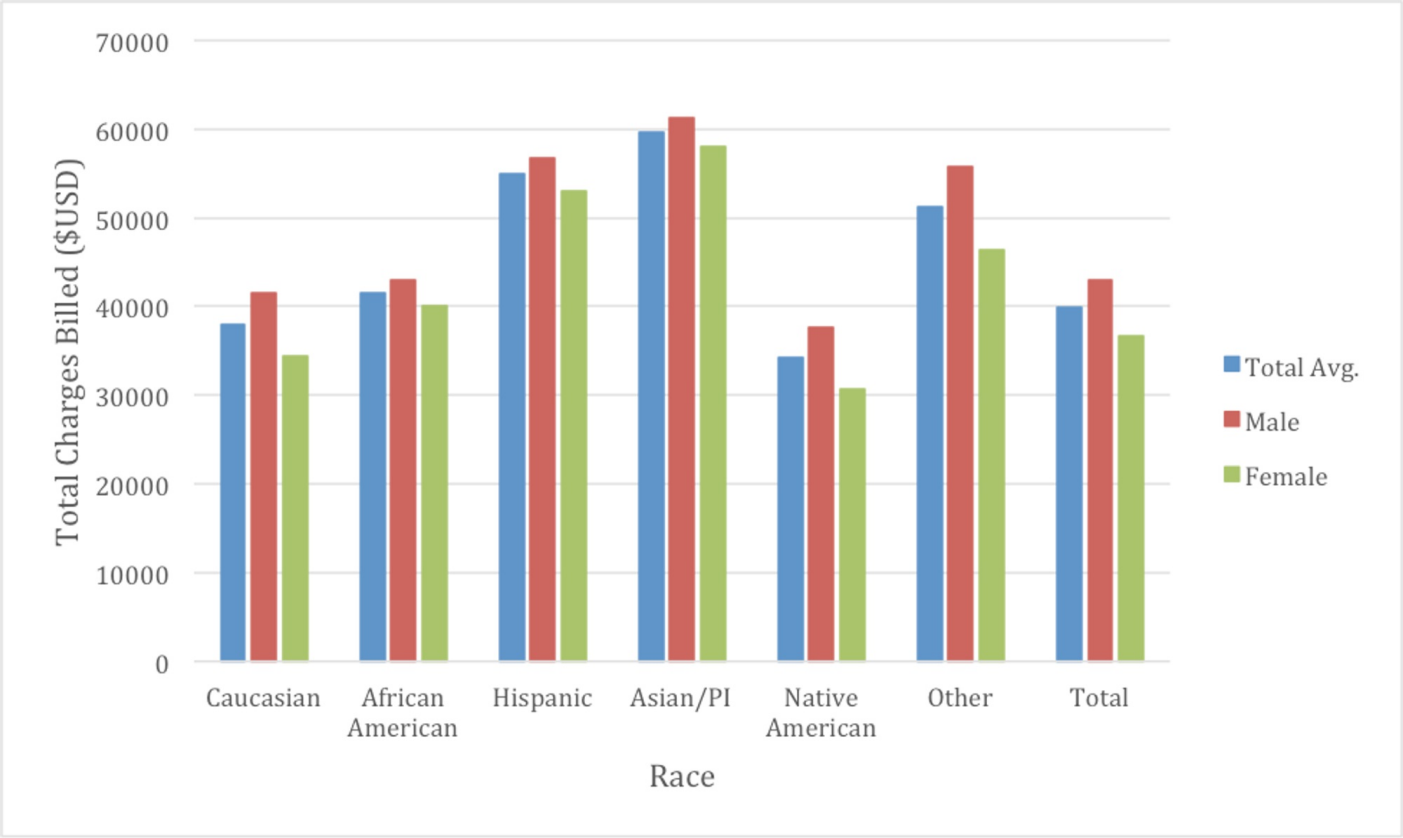
Total hospital charges billed by race and gender The total average cost billed over the study was $39847.43. The average cost for males was noted to be higher than females at $42920.90 vs $36744.69 (p<0.05). Native Americans were noted to have the least total charges billed on total average as well as by gender (p<0.05). Asians were noted to have the highest. The differences between the total average amongst races was noted to be statistically significant (p<0.05). Between genders, every race displayed gender differences in cost except for Asians which was trending to significance (p=0.06).

It was observed that Native Americans had an average LOS of 4.67 days which was significantly shorter than all other races (Figure 3). Caucasians had the second shortest LOS at 5.16 days, whereas the race specified as “Other” had the longest LOS at 5.67 days, exactly one day longer than that of Native Americans. When comparing the average total cost calculated as total charge to the patient/insurance company the results were surprising. Asians were noted to pay on average almost $25,000 more per CHF admission than compared to Native Americans ($59,668 vs $34,192, p < 0.05) (Figure 4). This is despite the fact that Asians had a LOS of only 0.49 days longer than Native Americans. When compared to Caucasians, Asians had a higher total charge of almost $22,000 ($59,668 vs $38,034 p <0.05). On multivariate analysis, the differences between races studied and the total charges billed were statistically significant at all levels of analysis between races. The type of insurance billed was also analyzed looking at Payor 1 and Payor 2 for hospital admission. Caucasians were the most likely to have Medicare as Payor 1 at 81.35% while they were also the most likely to have private insurance as Payor 2 at 54.08%. African Americans were the least likely to use Medicare as Payor 1 at 58.56% but most likely to use Medicaid as Payor 1 at 18.10%. Caucasians used Medicaid as Payor 1 at 3.80%.

**Figure 5:**
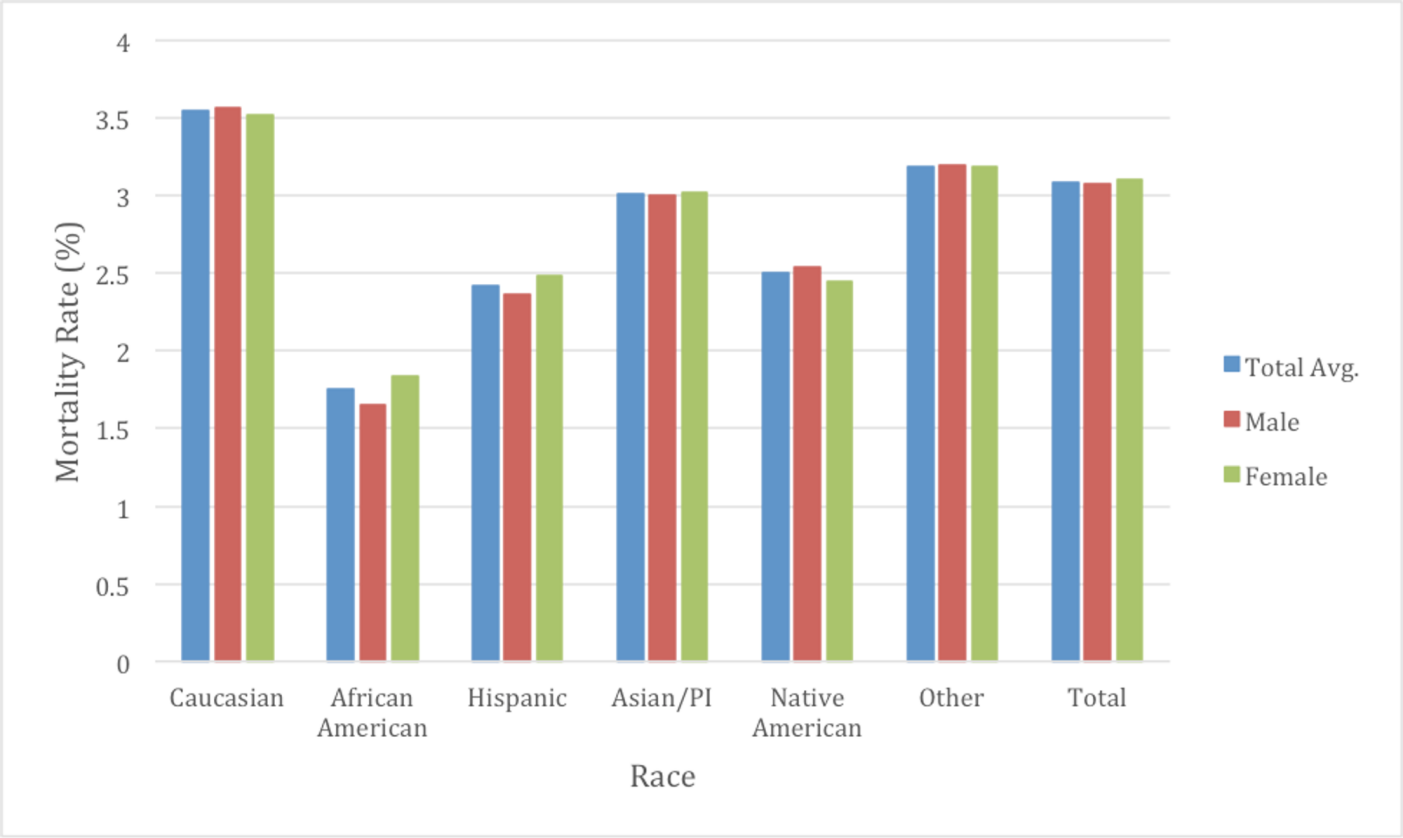
Mortality rate by race and gender The mortality rate was seen highest with Caucasians while African Americans were seen to have the lowest mortality. The differences amongst mortality amongst all the races were statistically significant (p<0.05). When comparing genders within each race, only African Americans displayed gender differences in mortality (p<0.05).

In terms of mortality, Caucasians were observed to have the highest mortality rate at 3.55% The race with the lowest mortality was African Americans at 1.75% (Figure 5). There was no significant difference between Native Americans and Hispanics in terms of mortality rates on multivariate analysis. Interestingly, Caucasians also had the highest average age at presentation at 75.76 while African Americans presented almost 12 years earlier in age at 63.47 and well below the average age amongst all races (p <0.05).

When comparing African Americans to Caucasians in regards to median household income quartile, it was found that Caucasians had the second highest income per ethnic group at 2.41 whereas African Americans had the lowest at 1.77. Asians were noted to have the highest income with a quartile of 2.87. The differences between all races for income quartile was statistically significant. The average income by quartile for all CHF admissions was approximately 2.25 over the five years studied. 

**Figure 6:**
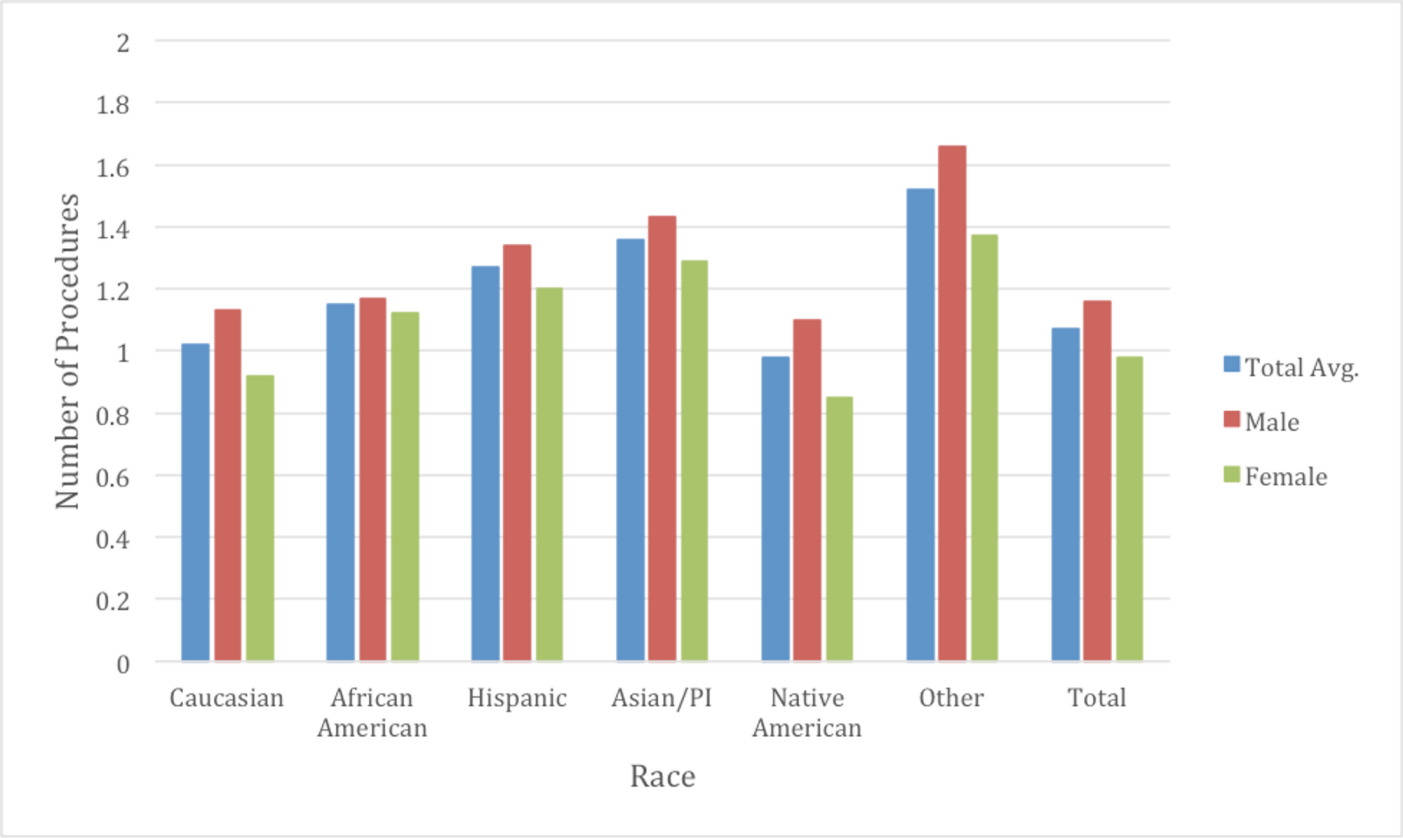
Number of procedures during congestive heart failure (CHF) admission by race and gender The average number of procedures during a CHF admission was 1.07. Native Americans were noted to have the least number of procedures done during their hospitalizations. Between genders, every race displayed gender differences in numbers of procedures (p<0.05).

The number of procedures during hospitalization (NPR) conducted during CHF admissions were also examined to help determine cost differences. The average NPR completed during the study in all races was approximately 1.08 (Figure 6). Native Americans had the least number of procedures completed during their hospitalizations at 0.98, while Asians had the second highest at 1.52 (p <0.05). Caucasians had an average of 1.02 number of procedures during their admissions. There was no statistical significance in the difference in NPR between Native Americans and Caucasians that was noted.

**Figure 7:**
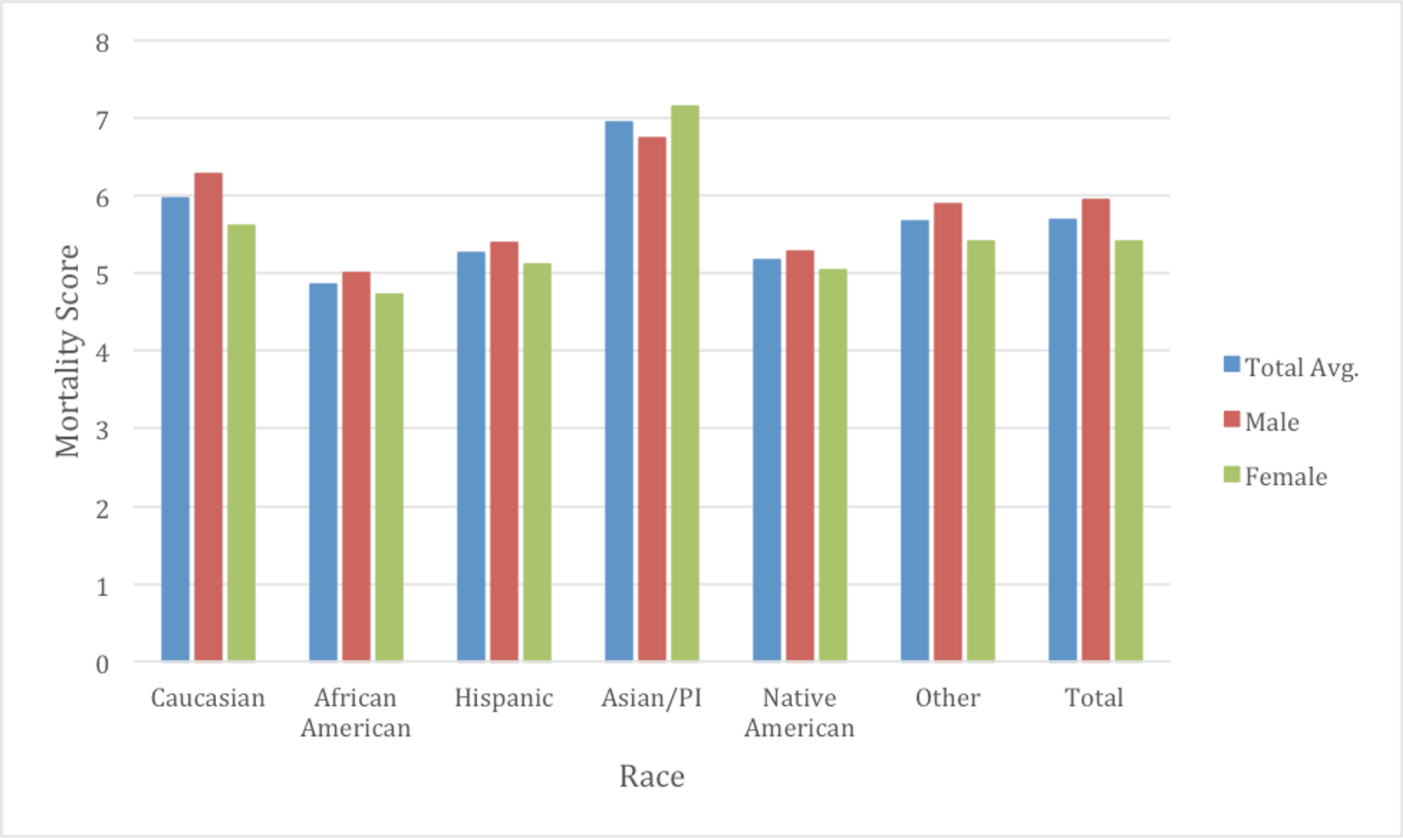
Elixhauser mortality score by race and gender The average Elixhauser mortality score was noted to be 5.69. Gender differences were also noted with males typically having a higher mortality score at 5.95 vs 5.42 for females (p<0.05). Between genders, every race displayed gender differences in cost except for Native Americans (p=0.39).

The Elixhauser mortality index scores were also analyzed. On average, the score increased from 5.17 in 2009 to 6.06 in 2014. African Americans were noted to have the lowest Elixhauser mortality score at 4.86, whereas Asians were noted to have the highest at 6.94 (p <0.05) (Figure 7). Caucasians had an average score of 5.96. On multivariate analysis between all races, statistical significance in differences was seen between all races except between Native Americans and Hispanics.

**Figure 8:**
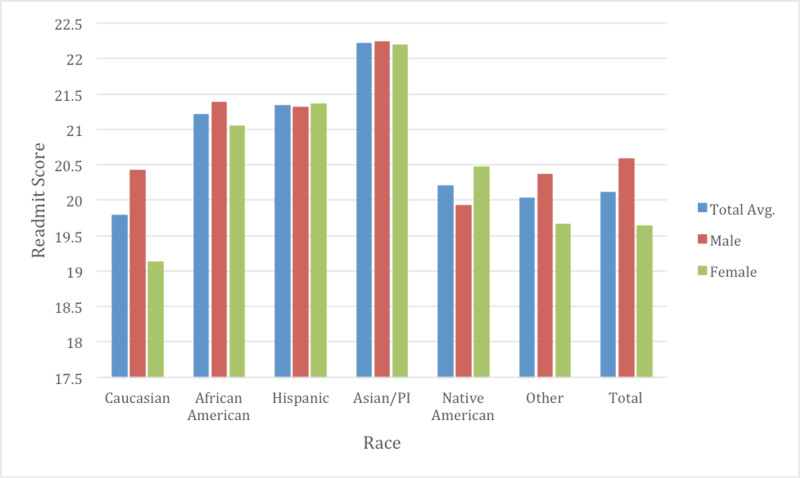
Elixhauser readmit score by race and gender The average Elixhauser readmit score was noted to be 20.11. Gender differences were also noted with males typically having a higher readmit score at 20.58 vs 19.64 for females (p<0.05). Between genders, only Caucasians, African Americans and “Other” were noted to have gender differences.

With the Elixhauser readmit score, the average score in 2009 was 18.42 which then rose to a level of 21.42 in 2014. Caucasians were seen to have the lowest score at 19.78 while Asians had the highest at 22.21 (p <0.05) (Figure 8). Only between Native Americans and Caucasians was there no statistical significance found on multi-variate analysis. Comorbidities were also analyzed based off the Elixhauser comorbidities (Table 1). Overall, the comorbidity with the highest prevalence in our study was shown to hypertension at 71.82% followed by renal failure at 38.50%, chronic lung disease at 36.39%, and diabetes without complications at 33.74%. The comorbidities seen least frequently were peptic ulcer disease at 0.03% followed by AIDS at 0.23%. On analysis as a whole, only peptic ulcer disease did not have any statistical significance noted between all races (p=0.18).

**Table 1:** Analysis and comparison of Elixhauser comorbidity prevalence (%) by race AIDS: acquired immunodeficiency syndrome, PVD: peripheral vascular disorders

Comorbid Condition	White	Black	Hispanic	Asian	Native American	Other	p-value
AIDS^a^	0.05	0.92	0.29	0.05	0.04	0.38	< 0.05
Valvular heart disease	0.40	0.32	0.34	0.51	0.17	0.40	< 0.05
Pulmonary circulation disorders	0.30	0.37	0.30	0.29	0.04	0.31	< 0.05
PVD^b^	12.57	8.42	11.79	9.25	9.33	9.77	< 0.05
Hypertension	69.42	80.18	76.72	75.45	68.37	73.29	< 0.05
Paralysis	1.54	2.33	2.03	3.08	1.35	1.70	< 0.05
Neurological disorders	7.52	5.29	5.76	5.17	4.73	6.00	< 0.05
Chronic pulmonary disease	38.22	33.72	30.57	26.69	32.28	32.57	< 0.05
Diabetes Mellitus (uncomplicated)	31.88	37.00	41.34	35.70	38.94	38.10	< 0.05
Diabetes Mellitus (Complicated)	8.66	10.24	16.16	15.56	11.89	10.73	< 0.05
Hypothyroidism	18.87	7.67	13.68	10.58	14.81	13.74	< 0.05
Renal failure	36.84	43.17	42.09	48.67	35.19	38.07	< 0.05
Liver disease	2.17	3.33	4.35	3.08	3.18	3.54	< 0.05
Peptic ulcer disease	0.03	0.03	0.04	0.05	0.04	0.04	0.18
Lymphoma	1.07	0.73	0.85	0.73	0.93	0.94	< 0.05
Metastatic cancer	1.10	0.75	0.74	0.99	0.67	0.82	< 0.05
Solid tumor without metastasis	1.88	1.45	1.23	1.26	1.05	1.35	< 0.05
Rheumatoid arthritis/Collagen vascular disease	2.84	2.56	2.09	2.20	2.61	2.23	< 0.05
Coagulopathy	4.94	4.17	5.21	7.23	4.23	5.22	< 0.05
Obesity	13.64	19.88	15.96	7.81	15.16	13.96	< 0.05
Weight loss	4.05	3.14	3.59	4.57	3.29	4.14	< 0.05
Electrolyte disorders	27.67	25.47	26.76	30.30	26.65	26.50	< 0.05
Chronic blood loss anemia	1.06	0.64	0.79	0.72	0.71	0.76	< 0.05
Deficiency anemias	28.32	29.95	32.81	34.72	26.91	29.71	< 0.05
Alcohol abuse	2.01	4.54	3.23	1.50	3.56	2.78	< 0.05
Drug abuse	0.97	6.73	2.45	1.82	1.89	2.21	< 0.05
Psychoses	2.64	3.52	2.84	1.78	2.02	2.53	< 0.05
Depression	10.30	5.66	7.51	4.42	7.77	7.34	< 0.05

Hypertension, one of the common risk factors associated with CHF was noted to have the highest prevalence in African Americans at 80.18% whereas the lowest prevalence was seen in Native Americans at 68.37% (p <0.05). Caucasians had the second lowest prevalence of hypertension at 69.42%. There was no significant difference between Caucasians and Native Americans for hypertension. Renal failure was observed highest in prevalence in Asians at 48.67% while the lowest was seen in Native Americans at 35.19% (p <0.05). Diabetes without complication was seen to be highest in prevalence in the Hispanic population at 41.34%. The least was within the Caucasian population at 31.88% (p <0.05). Obesity, considered a risk factor for ischemic cardiomyopathy, was seen highest in the African American population at 19.88% while the lowest was seen in Asians at 7.81% (p <0.05). The overall average prevalence of obesity was 14.83%.

Gender differences were also examined as a subgroup of races to determine if there were further healthcare inequalities (Table 2). The average age of males for CHF admissions was 70.3 while for females was approximately 75.1 years of age through the course of the study (Figure 2). Males typically had a higher total charge for their CHF admission with $38,702 in 2009 vs $32,472 with females, increasing to $47,586 in 2014 in males and $40,885 with females. The total average cost for males over the study was $42,920 and $36,744 for females (p <0.05). Mortality rate was observed at 3.08% in males and 3.10% in females (p =0.63) (Figure 5). LOS was slightly lower on average between males and females at 5.18 and 5.20 respectively (p <0.05) (Figure 3). Income quartile was seen to be slightly lower in females at 2.25 vs 2.26 in males (p <0.05). In terms of NPR, males had a greater number of procedures during their CHF admission at 1.16 vs 0.98 for females (p <0.05). Elixhauser mortality score was higher in males than females at 5.95 vs 5.42 (p <0.05). This was also true with the Elixhauser readmit score of 20.58 vs 19.64 (p <0.05). When comparing Elixhauser comorbidities, statistically significant gender differences were noted for all comorbidities except for peptic ulcer disease as well as for chronic lung disease with p-values of 0.50 and 0.23 respectively. The largest gender difference in terms of prevalence was noted with hypothyroidism with females seen at 21.66% and males at 10.13% (p <0.05). Hypertension was overall seen more prevalent in females at 73.26% vs 70.38% in males (p <0.05).

**Table 2:** Observed gender differences in age, total charges, length of stay, income quartile, number of procedures, as well as Elixhauser mortality score, readmission score and comorbidities Comorbid conditions presented as prevalence (%). All comorbidities were found to be statistically significant except for peptic ulcer disease and chronic lung disease. ZIPINC: Median household income for patient’s ZIP Code; NPR: Number of procedures during admission, AIDS: acquired immunodeficiency syndrome; PVD:peripheral vascular disorders.

	Males	Females	p-value
Age	70.30	75.12	<0.05
Total Charges ($)	42920.90	36744.69	<0.05
Length of Stay (days)	5.18	5.20	<0.05
ZIPINC^a^	2.26	2.25	<0.05
NPR^b^	1.16	0.98	<0.05
Readmission Score	20.58	19.64	<0.05
Mortality Score	5.95	5.42	<0.05
AIDS^c^	0.31	0.15	<0.05
Valvular heart disease	0.35	0.39	<0.05
Pulmonary circulation disorders	0.29	0.34	<0.05
PVD^d^	12.62	10.21	<0.05
Hypertension	70.38	73.26	<0.05
Paralysis	1.64	1.81	<0.05
Neurological disorders	6.04	7.56	<0.05
Chronic pulmonary disease	36.31	36.46	0.23
Diabetes Mellitus (uncomplicated)	34.02	33.47	<0.05
Diabetes Mellitus (Complicated)	9.99	9.32	<0.05
Hypothyroidism	10.13	21.66	<0.05
Renal failure	41.70	35.34	<0.05
Liver disease	3.22	1.91	<0.05
Peptic ulcer disease	0.03	0.03	0.5
Lymphoma	1.01	0.93	<0.05
Metastatic cancer	1.03	0.96	<0.05
Solid tumor without metastasis	2.10	1.32	<0.05
Rheumatoid arthritis/Collagen vascular disease	1.45	3.96	<0.05
Coagulopathy	5.73	3.94	<0.05
Obesity	14.01	15.63	<0.05
Weight loss	3.59	4.06	<0.05
Electrolyte disorders	25.31	28.94	<0.05
Chronic blood loss anemia	0.80	1.07	<0.05
Deficiency anemia	26.38	31.20	<0.05
Alcohol abuse	4.38	0.77	<0.05
Drug abuse	3.19	1.17	<0.05
Psychoses	2.54	3.04	<0.05
Depression	6.89	11.12	<0.05

Renal failure was more likely to be observed in males with CHF admissions at 41.70% whereas females were 35.34% (p <0.05).

On subgroup analysis between races and genders, the highest average age belonged to female Caucasians at 78.02 years, whereas the lowest was African American males at 60.76 years of age. Interestingly, almost the reverse was true in terms of mortality with African American males having the lowest mortality at 1.65% with Caucasian females at the second highest at 3.52% (p<0.05). Caucasian males overall had the highest mortality at 3.57%. Asian males had the highest total charges billed at $61,278 while Native American females had the lowest at $30,688. In terms of the Elixhauser mortality score, Asian females had the highest score at 7.15 and African American females had the lowest at 4.72 (p<0.05).

Even between each race, there were noted significant differences between the genders. Average age was noted to be statistically significant in terms of differences between genders no matter what ethnicity. Total charges were also noted to be almost similar with only Asians not showing a significant difference, but trending towards it (p=0.06). NPR was also noted to be statistically significant amongst genders in all races. Gender differences in mortality within race was only seen amongst African Americans with males at 1.65% and females at 1.84% (p <0.05).

Amongst the Elixhauser comorbidities, hypertension, hypothyroidism, liver failure, rheumatoid arthritis/collagen vascular disease, coagulopathy, anemia deficiency, alcohol use, drug use, and depression were all noted to be statistically significant between sexes for all races (p <0.05) (Table 3). Renal failure did not show a significant difference between males and females of Native American background at 34.61% and 35.74% (p = 0.72). All other races had gender differences for renal failure with males predominating (p <0.05). Out of all the comorbidities, only peptic ulcer disease as well as valvular heart disease did not show any significant differences between genders at any race. Otherwise, for every other comorbidity, there was at least one race-gender difference.

**Table 3:** Analysis of Elixhauser comorbidities between genders of each individual race Comorbid conditions presented as prevalence (%). AIDS: acquired immunodeficiency syndrome, PVD: peripheral vascular disorders

Comorbid Condtion	White			Black			Hispanic			Asian/PI			Native Amer			Other		
	Male	Female	p-value	Male	Female	p-value	Male	Female	p-value	Male	Female	p-value	Male	Female	p-value	Male	Female	p-value
AIDS	0.07	0.02	<0.05	1.16	0.69	<0.05	0.47	0.11	<0.05	0.04	0.06	0.1	0.07	0.00	0.99	0.56	0.19	<0.05
Valvular heart disease	0.39	0.40	0.2	0.28	0.36	0.07	0.31	0.37	0.49	0.46	0.55	0.57	0.14	0.20	0.75	0.35	0.45	0.54
Pulmonary circulation disorders	0.28	0.31	0.74	0.34	0.40	0.37	0.21	0.40	<0.05	0.34	0.23	0.98	0.08	0.00	0.32	0.24	0.38	<0.05
PVD\	14.17	10.99	<0.05	8.31	8.52	0.56	12.82	10.70	<0.05	9.86	8.62	0.45	11.04	7.69	<0.05	10.90	8.59	<0.05
Hypertension	67.76	71.06	<0.05	79.88	80.47	<0.05	74.89	78.65	<0.05	73.58	77.37	<0.05	66.22	70.43	<0.05	71.54	75.11	<0.05
Paralysis	1.48	1.60	<0.05	2.09	2.57	<0.05	2.03	2.03	0.51	2.84	3.33	0.07	1.40	1.30	0.98	1.50	1.91	0.9
Neurological disorders	6.61	8.42	<0.05	4.80	5.78	<0.05	4.99	6.57	<0.05	5.02	5.33	<0.05	4.36	5.09	<0.05	5.05	6.98	<0.05
Chronic pulmonary disease	38.82	37.63	<0.05	31.73	35.69	<0.05	29.69	31.50	<0.05	26.72	26.65	0.51	33.25	31.35	0.66	31.99	33.17	<0.05
Diabetes Mellitus (uncomplicated)	33.29	30.49	<0.05	34.10	39.87	<0.05	40.08	42.67	<0.05	35.00	36.42	0.99	38.50	39.36	0.21	37.13	39.11	0.1
Diabetes Mellitus (Complicated)	9.37	7.95	<0.05	9.41	11.06	<0.05	15.73	16.61	<0.05	16.16	14.94	0.23	11.43	12.33	0.28	10.71	10.75	0.29
Hypothyroidism	12.17	25.52	<0.05	4.40	10.91	<0.05	8.83	18.78	<0.05	6.87	14.38	<0.05	9.82	19.57	<0.05	8.65	19.02	<0.05
Renal failure	40.77	32.95	<0.05	45.53	40.82	<0.05	42.79	41.36	<0.05	50.96	46.33	<0.05	34.61	35.74	0.72	40.00	36.08	<0.05
Liver disease	2.68	1.66	<0.05	4.33	2.34	<0.05	5.52	3.13	<0.05	3.56	2.58	<0.05	4.42	1.99	<0.05	4.35	2.70	<0.05
Peptic ulcer disease	0.03	0.03	0.71	0.03	0.03	0.67	0.04	0.05	0.99	0.02	0.07	0.83	0.00	0.07	0.16	0.05	0.03	0.31
Lymphoma	1.14	0.99	<0.05	0.69	0.78	0.09	0.85	0.85	0.99	0.74	0.71	0.71	0.65	1.20	0.33	0.91	0.96	0.99
Metastatic cancer	1.17	1.04	<0.05	0.69	0.82	<0.05	0.69	0.80	0.07	1.10	0.87	0.46	0.76	0.58	0.84	0.95	0.68	0.19
Solid tumor without metastasis	2.32	1.44	<0.05	1.72	1.19	<0.05	1.48	0.97	<0.05	1.47	1.04	<0.05	1.27	0.83	0.18	1.71	0.97	<0.05
Rheumatoid arthritis/Collagen vascular disease	1.65	4.02	<0.05	1.04	4.07	<0.05	0.75	3.50	<0.05	1.10	3.34	<0.05	1.17	3.99	<0.05	1.26	3.23	<0.05
Coagulopathy	6.02	3.86	<0.05	4.59	3.76	<0.05	5.86	4.53	<0.05	7.57	6.88	<0.05	5.05	3.45	<0.05	6.18	4.23	<0.05
Obesity	13.62	13.65	0.11	16.58	23.15	<0.05	14.29	17.70	<0.05	8.14	7.46	<0.05	14.86	15.46	0.41	12.08	15.91	<0.05
Weight loss	3.76	4.32	<0.05	3.04	3.25	<0.05	3.31	3.89	<0.05	4.19	4.95	<0.05	3.11	3.47	0.41	3.93	4.35	0.17
Electrolyte disorders	25.70	29.62	<0.05	24.45	26.48	<0.05	24.89	28.73	<0.05	28.25	32.41	<0.05	24.31	28.90	<0.05	24.41	28.67	<0.05
Chronic blood loss anemia	0.97	1.15	<0.05	0.42	0.86	<0.05	0.64	0.95	<0.05	0.48	0.97	<0.05	0.48	0.93	0.14	0.70	0.82	0.27
Deficiency anemias	26.40	30.22	<0.05	26.43	33.43	<0.05	29.26	36.54	<0.05	30.77	38.78	<0.05	22.11	31.48	<0.05	26.56	32.96	<0.05
Alcohol abuse	3.40	0.64	<0.05	7.72	1.38	<0.05	5.80	0.54	<0.05	2.67	0.31	<0.05	5.77	1.46	<0.05	4.73	0.75	<0.05
Drug abuse	1.38	0.55	<0.05	9.84	3.65	<0.05	3.87	0.97	<0.05	2.68	0.93	<0.05	2.51	1.30	<0.05	3.48	0.91	<0.05
Psychoses	2.38	2.90	<0.05	3.21	3.83	<0.05	2.67	3.02	<0.05	1.73	1.84	<0.05	2.03	2.00	0.59	2.45	2.63	0.41
Depression	8.00	12.58	<0.05	4.06	7.24	<0.05	5.75	9.35	<0.05	3.51	5.35	<0.05	5.71	9.74	<0.05	4.93	9.84	<0.05

## Discussion

Chronic heart failure is one of the most common causes of inpatient stays nationally. According to the Healthcare Cost and Utilization project, it is the 4th most common reason for inpatient stay accounting for 297 stays per 100,000 [12]. It is typically a disease of the older population and can affect more than 10% of those older than 75 years [13]. According to the American Heart Association (AHA) we can expect the prevalence to continue to rise with the aging population. Despite the anticipated increase, our data shows the rates of hospitalizations for CHF have not increased and in fact, appeared to decrease over a six-year span. This may be attributed to improved outpatient management of heart failure, optimization of guideline-directed medical therapy, as well as the focus by CMS to decrease 30 readmissions [14]. Our data suggests, however, that this improvement in outpatient management and benefit from primary care may not be ubiquitous amongst the population. Herein, we will focus our discussion on the various health care disparities observed between ethnic groups.

Health care disparities are defined as “differences in health care quality, access, and outcomes adversely affecting members of racial and ethnic minority groups and other socially disadvantaged populations” [15]. Studies looking at these are becoming more abundant and rightfully so. The United States is projected to grow by 1.8 million per year between 2017 and 2060, with the majority of this growth coming from international migration than natural increase [16]. With an increasingly diverse population, studies on disparities amongst ethnic and racial groups become essential to identify pitfalls in our healthcare system to ensure quality and equality in the care that we deliver. These inequalities have been noted to occur in a variety of forms including patient’s experience of care, preventative care, and hospitalization [15]. 

Differences in heart failure exacerbations amongst various ethnicities have been previously reported using the NIS. Ziaeian et al. looked at heart failure hospitalizations and differences based on sex and race using the same database from years 2002 and 2013. Their study primarily focused on age-adjusted differences in hospitalization rates between ethnic groups [17]. The purpose of our study was to further investigate areas of inequality that pertain to their hospital course and cost.

The results of the study were quite staggering with multiple differences observed amongst ethnic groups. African American males and females were noted to be a particularly vulnerable population. Our study shows on average they present over a decade younger than their Caucasian counterparts. This is consistent with previous literature showing that African-Americans are 20 times more likely to have heart failure than whites in patients less than 50 years old [18]. African American patients are also shown to have a significantly higher readmission score but have a lower mortality score than white patients despite having a longer LOS and total charge. Lee et al. noted similar findings of increase LOS and risk of readmission, however, there were no differences in cost or mortality [19]. It is difficult to speculate the reason for a longer LOS despite better outcomes and lower mortality score. Differences may arise from type of payer as the African American population had a higher percentage of Medicaid as the primary payer. Further analysis is to be done to determine differences in disposition, and whether discharge home or to skilled nursing facilities may play a role in the LOS.

Readmission scores were significantly higher in minority populations with the exception of Native American. This may be in part to inequalities of chronic disease management that arise from previously demonstrated differences in health literacy, perceived discrimination, beliefs about medication, and non-adherence related to cost [15]. This may also be explained by differences in socioeconomic status. With the exception of the Asian population, our study shows minority populations present from lower income quartiles than Caucasian patients. Philbin et al. previously demonstrated in a large population of patients in acute-care hospitals that lower income was a positive predictor of readmission risk [20].

Perhaps the most shocking difference observed in our sample of patients is the large fluctuation of total charges amongst different ethnic groups. Specifically, Asian patients are observed to have a significant increase in total cost compared to all ethnic groups, with an impressive $25,000 difference from Native American counterparts. Indeed, when compared to Native Americans, Asian patients have a longer LOS by 0.66 days however, when we calculate the average daily cost of a CHF admission which our data approximates $9000, this only accounts for $6000. What about the other $19000? One potential explanation is that Asian population also had an increased mortality index therefore indicating more comorbidities which may drive the increase in hospital charges. A study in France demonstrated a “super-additive” increase in cost for chronic diseases in patients with comorbid conditions due to disease interaction [21]. Chen et al. analyzed in detail healthcare expenditure among Asian American subgroups. They specifically noted that expenditure on hospitalizations was not statistically significant compared to Caucasians for Chinese, Indian, and Asian populations. There was however a large increase in expenditure in the Other Asian group which unfortunately was not further specified [22]. Interestingly, it was demonstrated by Russo et al. that Asian/Pacific Islanders had the highest rate of patient safety events while in hospital, related to both surgical and medical complications [23]. Additionally, it may also be possible that due to the higher mortality score and the assumption these patients are more ill, that different medication and advanced therapies may have been offered resulting in increased total cost. In hospital complication rates have been linked to higher costs in previous studies. In an analysis of 64 different potentially preventable complications, 48 were observed to increase hospital cost by an additional estimated 9.4%-9.7% [24]. We also observed that Asians have more procedures done in hospital compared to other ethnicities, which may contribute to higher cost.

The gender differences were noted on multiple levels of our analysis. We found that on average, males were paying almost $6,200 more for CHF admissions than females were. Even with accommodating for the slightly longer LOS for females, males paid an average of $8,286 per day of admission vs $7,066 for females. This may potentially be explained by the fact that males were also seen to have more procedures done during their admission at 1.16 vs 0.98. What is interesting is that males appear to be more ill during their hospitalization based on their Elixhauser mortality score (5.95 vs 5.42 p <0.05), despite presenting at on average almost five years earlier than females. Not only did females present at a later age for CHF admissions, but that the mortality was seen to be similar between the two genders, further highlighting gender disparities. There have been mixed results from previous studies regarding gender differences in mortality. A retrospective analysis of the Candesartan in Heart Failure: Assessment of Reduction of Morbidity and Mortality (CHARM) program demonstrated significantly lower all cause mortality in women, as well as decreased risk of cardiovascular death and heart failure hospitalization [25]. Similarly, Parashar et al. showed a 15%-20% lower risk of all-cause and cardiovascular mortality in women [26]. Though there was no mortality difference in our study, we would have anticipated higher mortality in men as they were found to have more comorbidities as demonstrated by a higher Elixhauser Mortality Score. Perhaps one explanation is female patient’s presenting later in their disease course and have more severe heart failure. Our average age at presentation was greater than 70 for both males and females. A study of adults >65 years of age showed females used hospital services 21% less than men [27]. Furthermore, in a large study of almost 2 million hospitalizations, women were found to have less positive experience than men from issues arising communication, discharge information, and cleanliness [28]. Considering this, women may be less inclined to utilize hospital services with early signs and symptoms of worsening heart failure. Our study also shows females to have less procedures performed compared to males while inpatient. We did not further differentiate which procedures were performed but this raises the important question as to the etiology of heart failure and whether or not revascularization was required for ischemic etiologies. Sedlak et al. found that when adjusted for baseline differences, women were less likely to undergo cardiac catheterization than men when presenting with acute myocardial infarction [29]. This is an important consideration when over 60% of heart failure cases may have an underlying ischemic etiology [30].

Study limitation

With the use of a large all-payer database to conduct retrospective research, several limitations arise. Given that the data of comorbidities is deemed by ICD codes, it is possible that if diagnoses were not coded for that this may be underrepresented. Additionally, due to the NIS database being identified, there are likely instances of readmissions resulting in overrepresentation of certain factors. Also, due to the fact that medications and echocardiography results are not available, we are not truly able to determine the severity of heart failure and the contributions that gender and race may have on its development. This also can include the fact that we are unable to determine whether there were any stays within an intensive care unit or not, which would reflect increased costs charged to the patient. Furthermore, we only used primary diagnoses of CHF to eliminate the possibility of non-active CHF being incorporated into the study. The result of this is the possibility of eliminating active CHF in patients that were not coded as the primary issue during the hospitalization. Our purpose for this study was to determine the financial impact of race and gender disparities in CHF admissions.

## Conclusions

In conclusion, it is clear there are several disparities observed among different ethnic groups and between genders hospitalized for CHF exacerbation. The unfortunate reality is that these disparities are deeply rooted and multifaceted and have been shown to occur at the patient, provider, and systems level. There is likely no simple fix at this time, but using this study and the emerging data on healthcare disparities - especially with the increasingly diverse population of our nation - it is essential to design studies to further elucidate the source of these differences. Our goal as healthcare providers should be to continue to improve healthcare for all populations and promote cultural competency in efforts to minimize differences in patient experiences and the care we provide. 
